# Editorial: A Novel Approach to Plastic Surgery Journals: Thinking Out of the Mailbox

**Published:** 2008-05-28

**Authors:** Mark S. Granick

**Affiliations:** Professor of Surgery and Chief of Plastic Surgery, New Jersey Medical School-UMDNJ, Newark, New Jersey, USA

During the past year, 2 of the major plastic surgery journals have published editorials summarizing the various perceived roles of a plastic surgery journal. Rohrich suggested that the primary role is to represent plastic surgery societies as the “primary source for surgeons to keep current on the latest techniques, trends, and technologies.” Lineaweaver claimed that a journal, independent of any affiliated societies, should present a peer reviewed, selected “finite amount of information at regular intervals.” Both editors speculate on the role of Web-based journalism and mention open access (OA) publishing as a potential avenue for disseminating scientific information.

In fact, *ePlasty* is the embodiment of the OA mission. Our mission statement is the “*Open Access Journal of Plastic Surgery*…. Committed to the free exchange of knowledge in a global community.” My personal goal in establishing this journal was to bring current scientific information and educational resources to all plastic surgeons throughout the world without the traditional price and transportation barriers (Fig 1).

What exactly is “OA” publishing? How did it originate and what are the principles? My Co-Editor-in-Chief, Stephen Milner, MD, elaborated on these issues in a *Journal of Burns and Wounds* editorial in 2006. Essentially, OA publishing is paperless, digital, Web-based, free of charge, and devoid of copyright and licensing restrictions. Traditional publishing (TP) is a paper-restricted system, requires a complex distribution system, and is expensive for readers. Furthermore, authors do the work, write the papers, submit their graphics, illustrations and photos, and then sign over their copyright and licensing control to the publishers. OA publishing breaks down price barriers, allowing “free availability and unrestricted use” (open access overview). TP is costly to libraries and potential readers and establishes price barriers to access scientific literature (subscriptions, delivery fees, licensing fees, pay per view fees, reprint fees) and permission barriers (licensing and copyright limitations). OA publishing is Internet based, allowing immediate worldwide availability of publications. TP literature is paper bound and distribution is limited depending on the mail system in place. This may be relatively efficient if mailed within one country's postal boundaries (3–7 days). However, international mailing can delay delivery for weeks to months (Table 1). OA and TP are both compatible with all of the academic features of scholarly literature such as preservation of work, peer review, prestige, career advancement, and indexing.

Three major OA initiatives in Budapest (February 2002), Bethesda (June 2003), and Berlin (October 2003) form the common ground of the OA movement. The Budapest convention stated:

There are many degrees and kinds of wider and easier access to this literature. By “Open Access” to this literature, we mean its free availability on the public internet, permitting any users to read, download, copy, distribute, print, search, or link to the full texts of these articles, crawl them for indexing, pass them as data to software, or use them for any other lawful purpose, without financial, legal, or technical barriers other than those inseparable from gaining access to the internet itself. The only constraint on reproduction and distribution, and the only role for copyright in this domain, should be to give authors control over the integrity of their work and the right to be properly acknowledged and cited.

The Bethesda and the Berlin conferences stated that in OA publishing authors hold the copyright and must agree in advance to let readers “copy, use, distribute, transmit and display the work publicly and to make and distribute derivative works in any digital medium for any reasonable purpose, subject to proper attribution of authorship.” The importance of a free exchange of scholarly work is now recognized by the National Institutes of Health (NIH), which instituted a mandatory public access policy in April 2008. The NIH policy requires

that all investigators funded by the NIH submit or have submitted for them to the National Library of Medicine's PubMed Central an electronic version of their final peer-reviewed manuscripts upon acceptance for publication, to be made publicly available no later than 12 months after the official date of publication.

Both publishing systems require revenue to operate. However, the primary difference between OA and TP is that revenue in the OA system is not a barrier to readers. Authors publish their work in OA journals such as *ePlasty* without monetary expectation. Suber refers to this as “royalty-free literature.” Plastic surgeons do not write articles for dollars, but are motivated to advance knowledge accompanied by the expectation of prestige and career advancement. It is important to realize the OA literature is not free to produce or publish; it simply removes the price barriers to readers. Revenue is a requirement and various OA journals have adopted different models to raise operating funds. Some charge authors to publish. *ePlasty* has created an Exhibit Hall where sponsors can place multimedia descriptions of their products, link to their own Web sites, and exhibit much the same as they would at a large meeting. The Exhibit Hall has the potential to evolve into an industry-based depot of product descriptions and technical instructions, as well as a vehicle for introducing new technology.

The OA model provides for a repository of ideas, concepts, and all sorts of educational materials. *ePlasty* has elected to have an educational section extrinsic to the peer-reviewed journal. In the Education Section interesting cases, lectures, and multimedia presentations will be available to readers at no charge. A portion of this section will be devoted to program directors to facilitate their work. The CORE curriculum will evolve into a free “living textbook” of the essential knowledge base of plastic surgery. Additional free “living texts” will be introduced on other specific topics as well. In addition to all of these educational features, an interactive Forum is available for direct interchange of ideas, concepts, and questions. All of this is to promote a free exchange of knowledge for all plastic surgeons.

Medical publishing has slowly evolved over 400 years to its current TP mode; that is, until now. The digital era is upon us and OA literature is burgeoning due to the ability and need to share knowledge, experience, and expertise at a global level without the traditional constraints. The survival of our specialty is going to depend on the immediate free access by all plastic surgeons to the vast scholarly literature and wide array of educational resources. The worldwide community of plastic surgeons now has an OA vehicle in *ePlasty*. All are invited to participate.

## Figures and Tables

**Figure 1:**
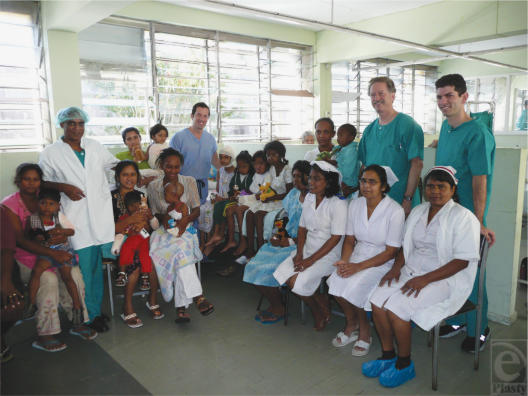
My team hosted by Dr R. P. Gunessee on the Plastic Surgery Ward at Victoria Hospital, Mauritius. Dr Gunessee is a well-trained plastic surgeon with limited access to the world's literature, but full access to the Internet.

**Table 1:** Comparison between open access (ePlasty) and paper-restricted systems (all other plastic surgery journals) of publication

Feature	ePlasty	Paper-restricted systems
Peer reviewed	+	±
PubMed indexed	+	±
PubMed Central indexed	+	−
Full text on PubMed	+	−
Free to readers	+	−
Free reprints	+	−
Authors own copyright	+	−
International audience (24 by 7 by 365)	+	−
Immediate world wide distribution	+	−
3-month submission to publication	+	−
Unlimited publishing space	+	−
Quality articles rejected due to space constraints	−	+
Immediate distribution of accepted articles	+	−
Scholarly impact	+	±
Environmentally self-sustaining	+	−
